# Trust in Doctors, Health Care System Distrust, and Cancer Screening Among Koreans

**DOI:** 10.3390/healthcare14091128

**Published:** 2026-04-23

**Authors:** Shin-Young Lee

**Affiliations:** Department of Nursing, Chosun University, Gwangju 61452, Republic of Korea; shinyoung0114@gmail.com

**Keywords:** trust, distrust, cancer, screening, Korean

## Abstract

**Background/Objectives**: Despite the availability of the National Cancer Screening Program in Korea, participation rates remain suboptimal. The literature demonstrates that cancer screening participation extends beyond individual-level knowledge and attitudes and is largely associated with trust. This study examines the role of trust—across cancer screening tests, health care providers, and health care organizations—as a central determinant of cancer screening participation among Koreans. **Methods**: A cross-sectional study was conducted with 369 Korean adults aged 40 years and older, recruited through convenience sampling from community centers in a metropolitan city. Data were collected using structured, paper-based questionnaires assessing socio-demographic factors and multilevel trust across specific screening tests, doctors, and health care organizations. Following descriptive statistics, bivariate and multivariate logistic regression analyses were performed to identify significant predictors of cancer screening utilization. **Results**: Koreans had relatively high trust in doctors and cancer screening tests. On an 11-point numeric rating scale ranging from 0 (not at all) to 10 (completely), the mean scores were 7.47 for the trust in doctors; colonoscopy had the highest trust score (M = 7.71), whereas the fecal occult blood test had the lowest (M = 7.14). Multivariate logistic regression revealed that trust and distrust were associated with the utilization of Pap smear, gastroscopy, upper gastrointestinal series, and colonoscopy in complex and sometimes paradoxical ways, and having a usual source of care was a consistent facilitator of cancer screening. **Conclusions**: These findings suggest that future research is needed to examine the complex interplay among trust, access to health care, and national policy in shaping cancer screening utilization in the Republic of Korea.

## 1. Introduction

Cancer is a life-threatening disease. It has remained the leading cause of death in the Republic of Korea for several decades, representing a critical public health challenge that threatens national population health [1,2]. Regarding cancer incidence and mortality in the Republic of Korea, the age-standardized cancer incidence rate increased from 518.0 per 100,000 population in 2019 to 522.9 per 100,000 population in 2023 [1,2,3]. Thyroid cancer was the most commonly diagnosed cancer in both 2019 and 2023, followed by lung, colorectal, breast, gastric, prostate, and liver cancers. In 2019, a total of 81,203 cancer deaths were reported, with a crude mortality rate of 158.2 per 100,000 population, accounting for approximately 27.5% of all deaths [3]. By 2024, cancer deaths increased to 88,933, with a crude mortality rate of 174.3 per 100,000 population, representing 24.8% of total deaths [1,2].

Although cancer remains a critical threat to life, early diagnosis of cancer significantly improves therapeutic outcomes [4,5,6]. Accordingly, regular cancer screening should be regarded as an important strategy for survival. The National Cancer Screening Program in the Republic of Korea is a nationwide, population-based screening initiative targeting cancers with high incidence and established early detection methods [7,8]. Initially launched in 1999 for low-income populations, the program was expanded in 2004 to include major cancers covering the entire eligible population [7]. Screening recommendations include biennial gastroscopy for gastric cancer in adults aged ≥40 years (with selective use of upper gastrointestinal series when endoscopy is not feasible), annual fecal occult blood testing for colorectal cancer in adults aged ≥50 years followed by colonoscopy if positive, biennial mammography for breast cancer in women aged ≥40 years, and biennial Pap smears for cervical cancer in women aged ≥20 years [7,8,9]. Overall, the National Cancer Screening Program is structured to promote early cancer detection through standardized, age-specific screening protocols while maintaining financial accessibility, with screening costs fully covered by the National Health Insurance Service [7,8].

The overall participation rate in the National Cancer Screening Program remained relatively low, with a slight increase from 55.6% in 2019 to 55.7% in 2024 [10]. Cancer-specific screening rates changed modestly, from 62.2% in 2019 to 62.5% in 2024 for gastric cancer, from 43.0% in 2019 to 41.1% for colorectal cancer, from 66.0% in 2019 to 65.2% for breast cancer, and from 59.8% in 2019 to 61.7% for cervical cancer [10]. There is an imperative need to enhance cancer screening participation rates to effectively reduce cancer incidence and mortality. This requires a systematic investigation into the behavioral patterns and determinants of cancer screening among the Korean population is warranted to develop evidence-based intervention strategies.

The existing literature indicates that cancer screening participation cannot be sufficiently explained by individual knowledge or attitudes alone but is strongly shaped by trust and distrust operating at multiple levels [11,12,13,14,15]. Trust in cancer screening operates across cancer screening tests, health care providers, and health care organizations. Previous studies [11,12,13,14,16,17,18] have demonstrated that trust in health care providers and the health care system is a powerful facilitator of screening participation, whereas distrust serves as a barrier to cancer screening utilization, and have emphasized trust as a core element cutting across factors associated with cancer screening utilization, including breast, cervical, and colorectal cancer screening across diverse ethnic populations. Therefore, trust in health care providers and the health care system is essential for the success of cancer screening programs [11,12,18].

Studies [14,19,20,21,22,23,24] involving Korean populations have reported similar findings regarding trust and cancer screening utilization, although the existing literature remains limited. For example, predictors of having a mammogram among Korean Americans in the United States were greater trust in health care providers, and lower distrust in the health care system [14]. Focus groups with Korean-speaking women in Sydney, Australia indicated that barriers to accessing breast cancer screening services were distrust in breast screening services [19]. Similarly, among Koreans in the Republic of Korea, although only a limited number of studies have examined trust in relation to cancer screening utilization, distrust toward the health care system and the National Cancer Screening Program has been repeatedly identified as a major barrier to cancer screening participation [20,21,24]. Qualitative studies have highlighted concerns regarding the accuracy of screening tests, perceptions of low-quality free screening services, and insufficient explanations from health care providers as central factors contributing to screening avoidance [20,21,22]. Distrust in the health care system refers to skepticism toward the free National Cancer Screening Program, with concerns that the equipment may be outdated or that examinations may be conducted perfunctorily; this distrust has been identified as one of the major reasons for avoiding cancer screening [20,21,22]. Koreans tend to place greater trust in university hospitals or large tertiary medical centers than in local clinics and place strong importance on quality of health care organizations [21,25]. To synthesize these findings, trust in cancer screening has been conceptualized as a multidimensional construct comprising trust in screening tests, health care providers, and health care organizations. These dimensions operate independently yet interactively to shape screening behaviors, particularly within government-led screening systems such as the Korean National Cancer Screening Program [21,25]. Accordingly, this study examines the role of trust—across cancer screening tests, health care providers, and health care organizations—as a central determinant of cancer screening participation among Koreans. By elucidating the role of trust as a core determinant of screening participation, the findings of this study are expected to provide empirical evidence to inform the development of effective, trust-based interventions aimed at improving cancer screening uptake and ultimately enhancing cancer survival in the Korean population.

## 2. Material and Methods

### 2.1. Study Design and Sample

**Figure 1:**
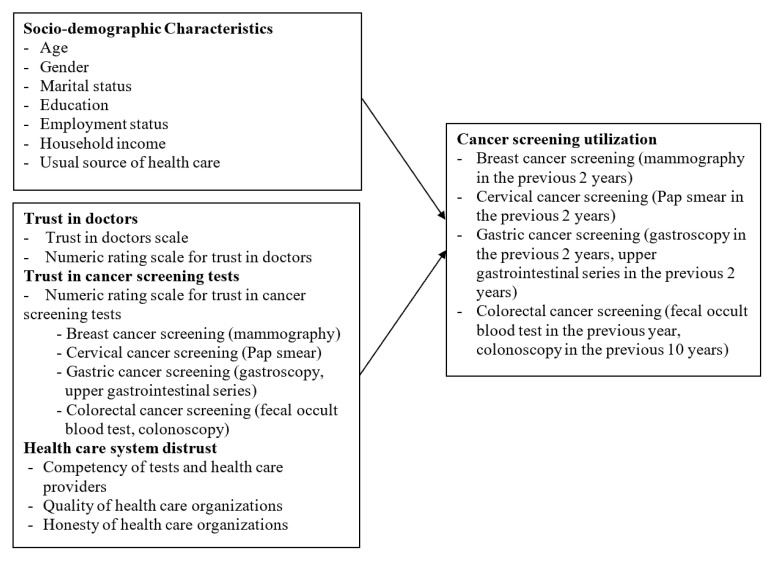
Conceptual Framework.

A cross-sectional study design was employed using a self-administered, paper-based questionnaire. The conceptual framework of this study is shown in Figure 1. The study sample consisted of Korean adults aged 40 years and older who were born in the Republic of Korea and were at average risk for cancer, defined as having no personal history of cancer and no first-degree family history of cancer based on the cancer screening guidelines [7]. A priori power analysis using G*Power (Version 3.1.9.7) [26] indicated a required sample size of 83 at an alpha level of 0.05 and a statistical power of 0.80, assuming a medium effect size derived from previous cancer screening studies and predictors included in the conceptual framework. The final sample size (N = 369) exceeded this requirement, indicating sufficient statistical power.

### 2.2. Data Collection

Participant recruitment was conducted using a convenience sampling strategy after approval from the Institutional Review Board of a university. The principal investigator (PI) visited community centers located in a metropolitan city in the Republic of Korea to recruit eligible participants. The PI explained the purpose of the study, procedures, potential risks, and confidentiality protections. All information on the study was provided in written form to potential participants. Participation was entirely voluntary. Individuals who expressed interest in participation contacted the PI and underwent an eligibility assessment using a standardized screening instrument approved by the Institutional Review Board (Approval No. 2-1041055-AB-N-01-2018-44). Participants were explicitly informed of their right to withdraw from the study at any time without penalty. All participants provided written informed consent prior to participation and completed a structured questionnaire requiring approximately 20 min. Of the 400 individuals initially recruited at community centers between June and August 2019, 369 completed the survey, yielding a response rate of 92.3%. To ensure anonymity and confidentiality, no personally identifiable information was collected, and all data were used exclusively for research purposes.

### 2.3. Measures

The survey questionnaire consisted of socio-demographic factors, multi-level trust (i.e., trust in doctors, trust in cancer screening tests, health care system distrust), trust in specific cancer screening tests (i.e., trust in mammography, Pap smear, gastroscopy, upper gastrointestinal series, fecal occult blood test, or colonoscopy), and participation in cancer screening. Socio-demographic factors measured age, gender, marital status, education, employment, household income, and usual source of health care (i.e., having a doctor or hospital for health screening regularly).

To measure trust in doctors, two scales were used in this study. First, the seven-item trust in doctors scale (e.g., I fully trust my physician regarding medical matters related to my health) was adapted from the trust scale of the Primary Care Assessment Survey [27]. Cronbach’s alpha for the trust in doctors scale was 0.86 [27]. Participants responded using a 5-point Likert scale ranging from 1 (strongly disagree) to 5 (strongly agree). Second, trust in doctors was also assessed using the numeric rating scale. Participants were asked, “All things considered, how much do you trust your doctor?” Responses were recorded on an 11-point numeric rating scale ranging from 0 (not at all) to 10 (completely), with higher scores indicating greater trust in doctors. The numeric rating scale is a widely used measurement approach for assessing subjective constructs in health research due to its simplicity and strong psychometric properties [28,29,30]. Previous studies have demonstrated that the numeric rating scale exhibits high test–retest reliability and strong agreement with multi-item scales, particularly when measuring global perceptions such as pain or trust [28,29,30].

Trust in specific cancer screening tests was assessed using the numeric rating scale as these constructs represent concrete and specific perceptions. Participants were asked to rate how much they trusted each screening test, including (a) breast cancer screening (mammography), (b) cervical cancer screening (Pap smear), (c) gastric cancer screening (gastroscopy and upper gastrointestinal series), and (d) colorectal cancer screening (fecal occult blood test and colonoscopy). Korean women were asked to respond to all items, including breast, cervical, gastric, and colorectal cancer screening, whereas Korean men were asked to respond only to items related to gastric and colorectal cancer screening. For each cancer screening test, participants responded to the following questions: “How much do you trust mammography?”, “How much do you trust the Pap smear?”, “How much do you trust gastroscopy?”, “How much do you trust the upper gastrointestinal series?”, “How much do you trust the fecal occult blood test?”, and “How much do you trust colonoscopy?” Responses were recorded on an 11-point numeric rating scale ranging from 0 (not at all) to 10 (completely). Higher scores indicated greater trust in the respective cancer screening test. Each trust score was analyzed separately to capture test-specific trust, reflecting participants’ perceptions of the accuracy and reliability of different screening procedures.

This study used a health care system distrust scale developed and validated through interviews, expert reviews, and a cross-sectional survey of 884 Koreans [25]. The health care system distrust scale for cancer screening [25] measured three subdomains: competency of tests and health care providers, quality of health care organizations, and honesty of health care organizations, reflecting Korean-specific experiences with cancer screening. The competency of tests and health care providers subdomain consisted of six items assessing perceptions of the competence of cancer screening tests and health care providers, including physicians and technicians [25]. The quality of health care organizations subdomain consisted of three items related to perceived differences in quality between hospitals and clinics, as well as between metropolitan hospitals and local hospitals [25]. The honesty of health care organizations subdomain consisted of three items assessing perceptions of truthfulness toward patients within health care settings [25]. The scale demonstrated adequate construct validity, supported by exploratory and confirmatory factor analyses, and showed good internal consistency, with Cronbach’s alpha coefficients ranged from 0.72 to 0.92 [25]. Participants responded using a 5-point Likert scale ranging from 1 (strongly disagree) to 5 (strongly agree). Higher scores indicated greater distrust in the competency of tests and health care providers, the quality of health care organizations, and the honesty of health care organizations.

Cancer screening utilization within the recommended intervals according to the National Cancer Screening Program guidelines in the Republic of Korea were measured in this study. Cancer screening tests included mammography in the previous 2 years, Pap smear in the previous 2 years, gastroscopy in the previous 2 years, upper gastrointestinal series in the previous 2 years, fecal occult blood test in the previous year, or colonoscopy in the previous 10 years. Each cancer screening utilization was measured using a question (e.g., When did you do your most recent mammography?). Participants indicated whether they had never undergone screening or reported the timing of their most recent screening test (e.g., less than 1 year).

### 2.4. Data Analysis

All statistical analyses were conducted using SPSS Version 30 [31]. No missing data were observed, as the PI verified questionnaire completeness at the time of data collection. Descriptive statistics, including means, standard deviations, frequencies, and percentages, were calculated to summarize socio-demographic characteristics and levels of trust in cancer screening tests, health care providers, and health care organizations. To examine the relationships between socio-demographic variables, trust in doctors, trust in cancer screening tests, health care system distrust, and cancer screening utilization among Koreans, a two-step analytic approach was employed. First, bivariate analyses were performed to assess the individual associations between each potential predictor and the utilization of specific cancer screening modalities (i.e., mammography, Pap smear, gastroscopy, upper gastrointestinal series, fecal occult blood test, and colonoscopy). Subsequently, variables that were statistically significant in the bivariate analyses (*p* < 0.05) were entered into multivariate logistic regression models. Bivariate screening using conventional significance thresholds has been commonly employed as an initial step to identify candidate variables while maintaining model parsimony and reducing the risk of overfitting, particularly in studies with moderate sample sizes [32]. Multivariate logistic regression analyses were then conducted to simultaneously evaluate the independent effects of all significant predictors on cancer screening utilization. Multicollinearity and model fit were assessed. Variance inflation factors (VIFs) were calculated for all independent variables. Model fit was evaluated using the Hosmer–Lemeshow goodness-of-fit test, and model discrimination was assessed using the area under the receiver operating characteristic curve (AUC).

## 3. Results

### 3.1. Sample Characteristics

Socio-demographic characteristics and cancer screening utilization of the sample are shown in Table 1. The mean age of the 369 Korean participants was 59.19 years. The majority of participants (87.0%) were married. More than half of the participants were women (56.9%) and did not have a usual source of care (51.8%). Regarding cancer screening utilization, more than half of the Korean women had undergone mammography in the previous 2 years (68.1%) and a Pap smear in the previous 2 years (74.8%). Among Korean men and women, 64.8% had undergone gastroscopy in the previous 2 years, 36.6% had undergone an upper gastrointestinal series in the previous 2 years, 49.6% had completed a fecal occult blood test in the previous year, and 69.6% had undergone colonoscopy in the previous 10 years.

### 3.2. Trust in Doctors, Cancer Screening Tests, and Health Care System Distrust

Table 2 presents the levels of trust in doctors, cancer screening tests, and health care system distrust. The mean score for the trust in doctors scale was 3.28, ranging from 2 to 5. On an 11-point numeric rating scale, the mean score was 7.47 for the trust in doctors. Trust mean scores for specific cancer screening tests were 7.38 for mammography, 7.35 for Pap smear, 7.67 for gastroscopy, 7.20 for the upper gastrointestinal series, 7.14 for fecal occult blood test, and 7.71 for colonoscopy. Colonoscopy had the highest trust score, whereas the fecal occult blood test had the lowest. The mean scores for health care system distrust were 2.76 for competency of tests and health care providers, 2.57 for quality of health care organizations, and 2.92 for honesty of health care organizations, on a scale ranging from 1 to 5. These findings indicate higher levels of distrust in the honesty of health care organizations and the competency of tests and health care providers.

### 3.3. Predictors of Cancer Screening

Bivariate logistic regression analyses identified several socio-demographic and trust-related factors associated with cancer screening utilization (Table 3). Mammography utilization in the previous 2 years was significantly associated with age, distrust in the competency of tests and health care providers, and distrust in the quality of health care organizations. Pap smear utilization in the previous 2 years was significantly associated with age, marital status, usual source of health care, trust in the Pap smear test, and distrust in the quality of health care organizations. Gastroscopy utilization in the previous 2 years was associated with having a usual source of health care and trust in gastroscopy, whereas upper gastrointestinal series utilization in the previous 2 years was associated with gender, usual source of health care, and trust in doctors. Fecal occult blood test utilization in the previous year was associated with employment status and having a usual source of health care. Colonoscopy utilization in the previous 10 years was associated with marital status, employment, having a usual source of health care, and distrust in the competency of tests and health care providers.

Variables that were statistically significant in the bivariate analyses above were entered into multivariate logistic regression models. Multicollinearity and model fit assessment indicated that no evidence of multicollinearity was observed and model fit was appropriate (all VIFs < 2.5). Multivariable logistic regression analyses identified significant predictors of cancer screening utilization among Koreans (Table 4). Age remained a significant predictor of mammography utilization, with women aged 40–64 years being nearly twice as likely to have undergone mammography in the previous 2 years compared with those aged 65 years and older (OR = 1.967, 95% CI = 1.016–3.810). For Pap smear utilization, both age and distrust in the quality of health care organizations were significant predictors. Korean women aged 40–64 years had more than twice the odds of Pap smear utilization compared with those aged 65 years and older (OR = 2.57, 95% CI = 1.25–5.29), while higher levels of distrust in the quality of health care organizations were associated with lower Pap smear utilization (OR = 0.648, 95% CI = 0.434–0.968). Regarding gastric cancer screening, gastroscopy utilization was independently associated with having a usual source of health care and trust in gastroscopy. Participants with a usual source of health care were more than twice as likely to undergo gastroscopy (OR = 2.720, 95% CI = 1.733–4.267), whereas higher trust in gastroscopy was significantly associated with lower utilization (OR = 0.854, 95% CI = 0.734–0.994). Upper gastrointestinal series utilization was significantly associated with gender, having a usual source of health care, and higher trust in doctors. Men, participants with a usual source of care, and those reporting higher trust in doctors were approximately 1.7–1.9 times more likely to have undergone upper gastrointestinal series screening (OR = 1.881, 95% CI = 1.193–2.967; OR = 1.809, 95% CI = 1.156–2.831; OR = 1.715, 95% CI = 1.006–2.923, respectively). Regarding colorectal cancer screening, fecal occult blood test utilization in the previous year was significantly associated with employment status and having a usual source of health care. Employed respondents were less likely to have undergone fecal occult blood test compared with unemployed individuals (OR = 0.559, 95% CI = 0.367–0.851), and having a usual source of health care nearly doubled the likelihood of fecal occult blood test utilization (OR = 1.944, 95% CI = 1.279–2.955). Colonoscopy utilization in the previous 10 years was significantly associated with marital status, employment, usual source of health care, and distrust in the competency of tests and health care providers. Married individuals and those with a usual source of health care were nearly twice as likely to have undergone colonoscopy, and higher distrust in the competency of tests and health care providers was also significantly associated with increased colonoscopy utilization (OR = 1.490, 95% CI = 1.089–2.040).

## 4. Discussion

This study examined the associations between trust in cancer screening tests, health care providers, and health care organizations on cancer screening behaviors among Koreans aged 40 years and older. Prior studies have typically focused on a single dimension of trust (e.g., physician trust or general health care system trust), whereas this study integrates multilevel trust constructs to better capture the complexity of factors associated with cancer screening behavior [11]. This study simultaneously examined multiple domains of trust—trust in cancer screening tests, trust in doctors, and health care system distrust—across multiple cancer screening tests—breast, cervical, gastric, and colorectal cancer screening—within a single conceptual framework. Furthermore, this study provides context-specific evidence within the Korean National Cancer Screening Program, where organized screening and institutional structures may shape the relationship between trust and cancer screening participation. Descriptive analyses in this study showed variation in cancer screening utilization rates and trust across cancer screening tests, along with relatively high levels of trust in doctors. First, the National Cancer Screening Program in the Republic of Korea primarily recommends mammography, Pap smear, gastroscopy, and fecal occult blood test. In this study, mammography, Pap smear, and gastroscopy utilization rates in the previous 2 years were more than 60% while fecal occult blood test in the previous year and upper gastrointestinal series in the previous 2 years were less than 40%. One of the reasons for low fecal occult blood test rates may be distrust in the cancer screening test. For example, a qualitative study with Koreans using individual interviews reported that participants distrusted the fecal occult blood test, which is a simple and basic test provided by the National Cancer Screening Program, and felt the need to undergo in-depth medical examinations such as colonoscopy to make sure it was the correct diagnosis [22]. In fact, the mean score of trust in cancer types was 7.14 for fecal occult blood test, and 7.71 for colonoscopy in this study, which were the lowest and highest trust scores, respectively, among cancer screening tests. Second, regarding trust in doctors, the mean score of trust in doctors was relatively high, 3.28 out of 5 and the mean score of trust in doctors item was 7.47 out of a 10 scale in this study. Although there are scarce studies measuring trust in doctors among Koreans, these trust scores were similar to a trust in doctors scale 3.10 out of 5 among Korean Americans [33] and a single-item measure of trust in health care provider yielded scores of 7.8 out of 10 in Korean Americans, 8.3 in Chinese Americans, 7.9 in Vietnamese Americans [34].

Trust was significantly associated with the utilization of Pap smear, gastroscopy, upper gastrointestinal series, and colonoscopy among Koreans, based on multivariate logistic regression analyses. Specifically, higher levels of distrust in the quality of health care organizations were associated with lower utilization of Pap smear screening (OR = 0.648, 95% CI = 0.434–0.968). Quality of health care organizations refers to the perception among Koreans that the quality of health care organizations varies across regional areas (local vs. metropolitan areas) and levels of health care organizations (small hospitals vs. large, general hospitals) [21,23]. Consequently, individuals who had greater distrust in the quality of hospitals—particularly regarding small and local facilities—were less likely to undergo a Pap smear test [21,23]. Furthermore, those with higher trust in doctors were approximately 1.7 times more likely to have undergone upper gastrointestinal series screening in this study (OR = 1.715, 95% CI = 1.006–2.923). While the upper gastrointestinal series is not the primary modality in the National Cancer Screening Program in the Republic of Korea, it is selectively used when gastroscopy is not feasible [7,8,9]. Gastroscopy is primarily recommended by the National Cancer Screening Program. Similar results on the association between trust in doctors, distrust in the health care system, and cancer screening were found in other studies: predictors of having a mammography were having a regular doctor or usual place for health care, greater trust in health care providers, and lower distrust in the health care system in Korean Americans [14], higher healthcare trust was associated with cervical cancer screening in United States populations [13], trust in doctors was associated with Pap smear test in African Americans [35] and distrust in the health care system was associated with Pap smear utilization in Latino immigrants in the United States [36].

Unexpectedly, higher trust in gastroscopy was significantly associated with lower gastroscopy utilization (OR = 0.85, 95% CI = 0.73–0.99). One possible explanation for this paradoxical finding is that the Korean government recommends several cancer screening tests at no or low cost, and gastroscopy is the primary modality recommended by the National Cancer Screening Program; therefore, Koreans may undergo gastroscopy despite low levels of trust in the test, health care providers, or the health care system. Under a plausible hypothesis, this pattern can be explained by the concept of obligation described in the context of the National Bowel Cancer Screening Program, a government-funded, population-based colorectal cancer program based on fecal occult blood test in Australia [17]. There are two types of obligation to participate in cancer screening: interpersonal obligation and institutional obligation [17]. Interpersonal obligation refers to a sense of responsibility toward one’s doctor, which may compel individuals to undertake the test in a timely manner, whereas institutional obligation arises from acknowledgment of the time and effort invested in program implementation, thereby encouraging participation in the national bowel cancer screening program [17]. Similarly, Koreans may have a sense of obligation to participate in the National Cancer Screening Program in the Republic of Korea, which may make weaken the associations between multilevel trust and cancer screening utilization. This obligation hypothesis needs to be examined in future studies to directly assess constructs such as perceived obligation or policy-driven compliance.

Another unexpected finding is that higher distrust in the competency of tests and health care providers including doctors or technicians was also significantly associated with increased colonoscopy utilization (OR = 1.490, 95% CI = 1.089–2.040). The health care system distrust scale for cancer screening measured perceptions of national cancer screening tests provided by the government, including the annual fecal occult blood test [23]. For example, items in competency of tests were “National cancer screening tests are not reliable because they are basic and simple”, “National cancer screening tests have a hard time detecting cancer because they are formally rough”, and “National cancer screening test results could be wrong, so I cannot believe them” [25]. These findings suggest that, regarding colorectal cancer screening, Koreans who reported higher distrust in the competency of fecal occult blood testing were more likely to undergo colonoscopy. The National Health Insurance Service offers basic cancer screening tests including fecal occult blood test which may be perceived as too simple to be fully trusted [21]. A lack of trust in the National Cancer Screening Program among Koreans has been reported, as some individuals believe that free screening services provided by the National Health Insurance Service are of lower quality [20,21]. Participants reported distrust in fecal occult blood test results and expressed concerns about the accuracy of the results [21]. This study demonstrated that the mean trust scores were 7.14 for fecal occult blood test, and 7.71 for colonoscopy. Accordingly, individuals may choose colonoscopy to obtain more trustworthy screening results for colorectal cancer. In addition, similar findings have been reported in the literature. For example, a recent study [37] reported that perceived benefits of fecal occult blood test showed no significant association with actual uptake, indicating that fecal occult blood test participation may be largely influenced by its mandated inclusion in the National Cancer Screening Program. Korean adults demonstrate lower confidence in the diagnostic reliability of fecal occult blood test compared with colonoscopy, as reflected in higher recognition rates and perceived benefit scores for colonoscopy, indicating a prevailing belief in its superior diagnostic effectiveness [37]. These findings underscore the need to enhance the perceived credibility and diagnostic reliability of cancer screening tests to strengthen public trust in the National Cancer Screening Program among Koreans.

Among the variables examined, having a usual source of health care was another significant predictor of several cancer screening modalities, including gastric cancer screening (i.e., gastroscopy and upper gastrointestinal series), and colorectal cancer screening (i.e., fecal occult blood test and colonoscopy). Specifically, participants with a usual source of health care were more than twice as likely to undergo gastroscopy (OR = 2.720), upper gastrointestinal series (OR = 1.809), fecal occult blood test (OR = 1.944), and colonoscopy (OR = 1.794). The data suggest that fostering a regular source of health care is vital for improving cancer screening adherence among the target population. Having a usual source of care (i.e., having a doctor or hospital for health screening regularly) may be related to trust in screening tests and health care providers. A total of 41 systematic reviews [12] indicated that, across all factors that influence the uptake of breast, cervical, and colorectal cancer screening, trust and building trusted relationships are integral to the success of cancer screening program. Strengthening the credibility and influence of health care providers should be a central priority, given that trust in a provider’s recommendation is a major facilitator of cancer screening participation, whereas distrust toward the provider administering the test constitutes a substantial barrier to screening uptake [12]. Additionally, Koreans have reported perceived barriers to cancer screening, including distrust in screening tests and negative experiences with health care providers; for example, busy doctors or nurses who did not provide detailed explanations of test procedures and results discouraged participation in screening [21]. To build trust in screening tests and health care providers, health care settings and policies should prioritize clear communication and detailed explanations of screening procedures and results.

This study contributes to the literature by identifying associations between trust in cancer screening tests, health care providers, and health care organizations, and cancer screening utilization within the context of the National Cancer Screening Program implemented by the Korean government, representing a novel contribution to the emerging body of knowledge on multilevel trust and cancer screening utilization. Despite this contribution, several limitations warrant caution when interpreting these results. First, the use of voluntary convenience sampling may introduce selection bias and restrict the generalizability of the findings. Recruiting through community centers might have led to an overrepresentation of individuals with high levels of social engagement or health awareness compared to the general population. As a result, cancer screening participation rates and levels of trust observed in this study may be overestimated. Second, the reliance on self-reported measures introduces the potential for response bias. Notably, participants’ recollection of specific dates is susceptible to recall bias, which may result in measurement errors or misclassification. These sources of inaccuracy may have affected the estimated relationships and should be considered when interpreting the study findings. Third, variables with *p*-values less than 0.05 in the bivariate analyses were selected for inclusion in the multivariable logistic regression models in this study. The use of statistical significance thresholds in variable selection may not fully account for all potential confounding effects. Fourth, the use of different timeframes across cancer screening modalities (e.g., mammography in the previous 2 years or colonoscopy in the previous 10 years) based on the national cancer screening guidelines in Korea may limit direct comparability and interpretation. Also, colonoscopy participation over a 10-year period may introduce temporal ambiguity when examining its association with trust measured at a single time point, as trust measured at the time of survey may not reflect trust at the time the colonoscopy was performed.

## 5. Conclusions

This study highlights that multilevel trust—specifically in cancer screening tests, health care providers, and health care organizations—is a critical yet complex determinant of cancer screening participation in the Republic of Korea. While a usual source of care consistently facilitates screening, paradoxical relationships between trust and certain cancer screening tests suggest that the National Cancer Screening Program’s mandated structure may attenuate the influence of individual perceptions of reliability. These findings indicate that future research is needed to examine the complex interplay among trust, access to health care, and national policy in shaping cancer screening utilization in the Republic of Korea.

## Figures and Tables

**Table 1:** Socio-demographic and Cancer Screening Utilizations among Koreans (N = 369).

Variable	n (%)	M ± SD	Range
**Socio-demographics**			
Age (year)			
40–64	257 (69.6)	59.19 ± 8.20	40–79
≥65	112 (30.4)		
Gender			
Male	159 (43.1)		
Female	210 (56.9)		
Marital status			
Currently married	321 (87.0)		
Not married	48 (13.0)		
Education			
≤High school graduate	188 (50.9)		
>High school graduate	181 (49.1)		
Employment			
Employed	201 (54.5)		
Unemployed	168 (45.5)		
Household income			
≤$4000	223 (60.6)		
>$4000	145 (39.4)		
Usual source of health care			
Yes	178 (48.2)		
No	191 (51.8)		
**Cancer screening utilization**			
Breast cancer screening			
Mammography in the previous 2 years (N = 210)			
Yes	143 (68.1)		
No	67 (31.9)		
Cervical cancer screening			
Pap smear in the previous 2 years (N = 210)			
Yes	157 (74.8)		
No	53 (25.2)		
Gastric cancer screening			
Gastroscopy in the previous 2 years			
Yes	239 (64.8)		
No	130 (35.2)		
Upper gastrointestinal series in the previous 2 years			
Yes	135 (36.6)		
No	234 (63.4)		
Colorectal cancer screening			
Fecal occult blood test in the previous year			
Yes	183 (49.6)		
No	186 (50.4)		
Colonoscopy in the previous 10 years			
Yes	257 (69.6)		
No	112 (30.4)		

Note. M = mean; SD = Standard deviation.

**Table 2:** Trust in doctors, screening tests, and health care system distrust (N = 369).

Trust	M ± SD	Range
**Trust in doctors**		
Trust in doctors scale	3.28 ± 0.48	2–5
Numeric rating scale for trust in doctors	7.47 ± 1.58	2–10
**Trust in cancer screening tests**		
Breast cancer screening (N = 210)		
Mammography	7.38 ± 1.66	2–10
Cervical cancer screening (N = 210)		
Pap smear	7.35 ± 1.63	3–10
Gastric cancer screening		
Gastroscopy	7.67 ± 1.51	3–10
Upper gastrointestinal series	7.20 ± 1.74	1–10
Colorectal cancer screening		
Fecal occult blood test	7.14 ± 1.81	0–10
Colonoscopy	7.71 ± 1.50	2–10
**Health care system Distrust**		
Competency of tests and health care providers	2.76 ± 0.75	1–5
Quality of health care organizations	2.57 ± 0.77	1–5
Honesty of health care organizations	2.92 ± 0.74	1–5

Note. M = mean; SD = Standard deviation.

**Table 3:** Factors Associated with Cancer Screening from Bivariate Logistic Regression.

	Mammography (N = 210)				Pap Smear (N = 210)			
	B	SE	OR (95% CI)	*p*-Value	B	SE	OR (95% CI)	*p*-Value
**Socio-demographics**								
Age (year)								
40–64 (ref. ≥ 65)	0.646	0.320	1.908 (1.019–3.573)	0.043	1.059	0.336	2.885 (1.493–5.574)	0.002
Marital status								
Currently married	0.657	0.374	1.929 (0.926–4.020)	0.079	0.939	0.385	2.556 (1.203–5.433)	0.015
Education								
>High school graduate	0.188	0.302	1.207 (0.668–2.182)	0.534	−0.082	0.321	0.922 (0.491–1.729)	0.799
Employment								
Employed	−0.096	0.296	0.908 (0.508–1.623)	0.745	0.330	0.321	1.391 (0.741–2.577)	0.304
Household income								
>$40,000	0.174	0.317	1.190 (0.640–2.214)	0.582	0.231	0.343	1.259 (0.643–2.466)	0.501
Usual source of health care	0.523	0.307	1.686 (0.924–3.079)	0.089	0.840	0.344	2.317 (1.180–4.550)	0.015
**Trust**								
Trust in doctors scale	0.523	0.324	1.687 (0.893–3.186)	0.107	0.339	0.344	1.403 (0.716–2.751)	0.324
Numeric rating scale for trust in doctors	0.049	0.093	1.050 (0.874–1.261)	0.601	−0.118	0.104	0.888 (0.725–1.088)	0.253
Trust in specific cancer screening	−0.179	0.095	0.836 (0.694–1.007)	0.059	−0.222	0.107	0.801 (0.649–0.988)	0.038
**Health care system distrust**								
Competency	−0.416	0.191	0.660 (0.453–0.959)	0.029	−0.336	0.201	0.715 (0.482–1.060)	0.095
Quality	−0.433	0.182	0.648 (0.454–0.926)	0.017	−0.436	0.193	0.646 (0.443–0.943)	0.023
Honesty	−0.208	0.194	0.812 (0.556–1.187)	0.283	−0.123	0.207	0.884 (0.590–1.326)	0.552
	**Gastroscopy (N = 369)**				**Upper gastrointestinal series (N = 369)**			
	**B**	**SE**	**OR (95% CI)**	***p*** **-Value**	**B**	**SE**	**OR (95% CI)**	***p*** **-Value**
**Socio-demographics**								
Age (year)								
40–64 (ref. ≥ 65)	0.307	0.234	1.359 (0.859–2.150)	0.190	−0.382	0.232	0.682 (0.433–1.075)	0.100
Gender								
Female	0.385	0.220	1.470 (0.956–2.261)	0.080	0.542	0.223	1.719 (1.110–2.662)	0.015
Marital status								
Currently married	0.512	0.313	1.669 (0.904–3.081)	0.102	−0.345	0.313	0.708 (0.383–1.309)	0.271
Education								
>High school graduate	0.371	0.219	1.449 (0.943–2.227)	0.091	0.224	0.216	1.251 (0.818–1.912)	0.302
Employment								
Employed	−0.105	0.219	0.900 (0.586–1.384)	0.632	−0.166	0.217	0.847 (0.554–1.295)	0.443
Household income >$40,000	0.263	0.226	1.301 (0.836–2.024)	0.244	0.206	0.221	1.228 (0.797–1.893)	0.352
Usual source of health care	1.018	0.229	2.767 (1.768–4.330)	<0.001	0.511	0.218	1.667 (1.088–2.555)	0.019
**Trust**								
Trust in doctors scale	0.385	0.231	1.469 (0.357–2.309)	0.095	0.716	0.233	2.047 (1.296–3.234)	0.002
Numeric rating scale for trust in doctors	0.039	0.069	1.040 (0.909–1.191)	0.567	0.190	0.072	1.209 (1.050–1.393)	0.008
Trust in specific cancer screening	−0.171	0.076	0.843 (0.726–0.978)	0.025	0.076	0.063	1.079 (0.953–1.221)	0.232
**Health care system distrust**								
Competency	−0.087	0.145	0.917 (0.690–1.218)	0.549	−0.003	0.144	1.003 (0.757–1.331)	0.981
Quality	−0.178	0.141	0.837 (0.635–1.103)	0.205	−0.184	0.142	0.832 (0.629–1.100)	0.197
Honesty	−0.001	0.148	0.999 (0.747–1.336)	0.995	−0.091	0.147	0.913 (0.684–1.219)	0.537
	**Fecal occult blood test (N = 369)**				**Colonoscopy (N = 369)**			
	**B**	**SE**	**OR (95% CI)**	***p*** **-value**	**B**	**SE**	**OR (95% CI)**	***p*** **-value**
**Socio-demographics**								
Age (year)								
40–64 (ref. ≥ 65)	−0.229	0.227	0.796 (0.510–1.241)	0.313	0.000	0.246	1.000 (0.617–1.620)	0.999
Gender								
Female	0.170	0.210	1.186 (0.785–1.791)	0.418	−0.385	0.233	0.680 (0.431–1.073)	0.098
Marital status								
Currently married	0.270	0.312	1.310 (0.711–2.413)	0.386	0.875	0.315	2.398 (1.294–4.444)	0.005
Education								
>High school graduate	−0.077	0.208	0.926 (0.616–1.393)	0.713	0.358	0.228	1.430 (0.915–2.236)	0.117
Employment								
Employed	−0.558	0.211	0.566 (0.375–0.856)	0.007	−0.472	0.232	0.624 (0.396–0.982)	0.042
Household income								
>$40,000	−0.352	0.214	0.703 (0.462–1.070)	0.100	0.335	0.236	1.398 (0.880–2.223)	0.156
Usual source of health care	0.645	0.211	1.905 (1.260–2.882)	0.002	0.682	0.233	1.977 (1.253–3.119)	0.003
**Trust**								
Trust in doctors scale	0.131	0.218	1.140 (0.745–1.747)	0.546	−0.280	0.237	0.756 (0.475–1.202)	0.237
Numeric rating scale for trust in doctors	0.015	0.066	1.015 (0.892–1.155)	0.819	−0.006	0.072	0.994 (0.864–1.145)	0.936
Trust in specific cancer screening	−0.056	0.058	0.946 (0.844–1.059)	0.334	−0.030	0.076	0.971 (0.836–1.127)	0.696
**Health care system distrust**								
Competency	0.065	0.139	1.068 (0.813–1.402)	0.638	0.311	0.155	1.364 (1.007–1.848)	0.045
Quality	0.016	0.135	1.016 (0.780–1.324)	0.907	0.198	0.150	1.219 (0.909–1.636)	0.187
Honesty	0.159	0.142	1.172 (0.886–1.549)	0.265	0.211	0.155	1.235 (0.910–1.674)	0.175

Note. OR = Odds ratio; CI = Confidence interval.

**Table 4:** Factors Significantly Associated with Cancer Screening Utilization from Multivariate Logistic Regression (N = 369).

	B	SE	OR (95% CI)	*p*-Value
**Mammography** (N = 210)				
Age 40–64 (ref. ≥ 65)	0.677	0.337	1.967 (1.016–3.810)	0.045
Chi-square likelihood ratio test = 10.873, df = 3, *p*-value < 0.05				
**Pap smear** (N = 210)				
Age 40–64 (ref. ≥65)	0.944	0.368	2.569 (1.248–5.288)	0.010
Quality of health care organizations (Health care system distrust)	−0.433	0.205	0.648 (0.434–0.968)	0.034
Chi-square likelihood ratio test = 22.112, df = 5, *p*-value < 0.001				
**Gastroscopy**				
Usual source of health care (ref. No)	1.000	0.230	2.720 (1.733–4.267)	<0.001
Trust in gastroscopy	−0.157	0.077	0.854 (0.734–0.994)	0.041
Chi-square likelihood ratio test = 25.043, df = 2, *p*-value < 0.001				
**Upper gastrointestinal series**				
Female (ref. Male)	0.632	0.232	1.881 (1.193–2.967)	0.007
Usual source of health care (ref. No)	0.593	0.228	1.809 (1.156–2.831)	0.009
Trust in doctors scale	0.540	0.272	1.715 (1.006–2.923)	0.047
Chi-square likelihood ratio test = 24.598, df = 4, *p*-value < 0.001				
**Fecal occult blood test**				
Employment (ref. No)	−0.581	0.214	0.559 (0.367–0.851)	0.007
Usual source of health care (ref. No)	0.665	0.214	1.944 (1.279–2.955)	0.002
Chi-square likelihood ratio test = 16.889, df = 2, *p*-value < 0.001				
**Colonoscopy**				
Marital status (ref. No)	0.849	0.337	2.336 (1.061–4.526)	0.012
Employment (ref. No)	−0.520	0.239	0.594 (0.372–0.949)	0.029
Usual source of health care (ref. No)	0.585	0.244	1.794 (1.113–2.892)	0.016
Competency of tests and health care providers (Health care system distrust)	0.399	0.160	1.490 (1.089–2.040)	0.013
Chi-square likelihood ratio test = 24.256, df = 4, *p*-value < 0.001				

Note. OR = Odds ratio; CI = Confidence interval; Ref. = Reference.

## Data Availability

All the data are available from the corresponding author upon reasonable request due to privacy restrictions.
